# Assessment of Chemical Impact of Invasive Bryozoan *Pectinatella magnifica* on the Environment: Cytotoxicity and Antimicrobial Activity of *P. magnifica* Extracts

**DOI:** 10.3390/molecules21111476

**Published:** 2016-11-04

**Authors:** Peter Kollar, Karel Šmejkal, Hana Salmonová, Eva Vlková, Olga Lepšová-Skácelová, Zuzana Balounová, Josef Rajchard, Josef Cvačka, Libor Jaša, Pavel Babica, Jiří Pazourek

**Affiliations:** 1 Department of Human Pharmacology and Toxicology, Faculty of Pharmacy, University of Veterinary and Pharmaceutical Sciences, Palackého tř. 1946/1, Brno 61242, Czech Republic; 2 Department of Natural Drugs, Faculty of Pharmacy, University of Veterinary and Pharmaceutical Sciences, Palackého tř. 1946/1, Brno 61242, Czech Republic; karel.mejkal@post.cz; 3 Department of Microbiology, Nutrition and Dietetics, Faculty of Agrobiology, Food and Natural Resources, Czech University of Life Sciences Prague, Kamýcká 129, Prague 6, 16521, Czech Republic; salmonova@af.czu.cz (H.S.); vlkova@af.czu.cz (E.V.); 4 Department of Botany, Faculty of Science, University of South Bohemia in České Budějovice, Branišovská 31, České Budějovice 37005, Czech Republic; oskacelova@prf.jcu.cz; 5 Department of Biological Studies, Faculty of Agriculture, University of South Bohemia in České Budějovice, Studentská 13, České Budějovice 37005, Czech Republic; baloun@zf.jcu.cz (Z.B.); rajchard@zf.jcu.cz (J.R.); 6 Institute of Organic Chemistry and Biochemistry, Academy of Sciences of the Czech Republic, v.v.i., Flemingovo nám. 2, Prague 16610, Czech Republic; josef.cvacka@uochb.cas.cz; 7 RECETOX—Research Centre for Toxic Compounds in the Environment, Faculty of Science, Masaryk University, Kamenice 753/5, Brno 60200, Czech Republic; jasa@recetox.muni.cz (L.J.); babica@recetox.muni.cz (P.B.); 8 Department of Experimental Phycology and Ecotoxicology, Institute of Botany, Academy of Sciences of the Czech Republic, Lidická 25/27, Brno 60200, Czech Republic; 9 Department of Chemical Drugs, Faculty of Pharmacy, University of Veterinary and Pharmaceutical Sciences, Palackého tř. 1946/1, Brno 61242, Czech Republic; pazourekj@vfu.cz

**Keywords:** *Aeromonas*, antimicrobial activity, bacteria, Bryozoa, cyanobacteria, invasive species, *Pectinatella magnifica*, toxicity

## Abstract

*Pectinatella magnifica*, an invasive bryozoan, might significantly affect ecosystem balance due to its massive occurrence in many areas in Europe and other parts of the world. Biological and chemical analyses are needed to get complete information about the impact of the animal on the environment. In this paper, we aimed to evaluate in vitro cytotoxic effects of five extracts prepared from *P. magnifica* using LDH assay on THP-1 cell line. Antimicrobial activities of extracts against 22 different bacterial strains were tested by microdilution method. Our study showed that all extracts tested, except aqueous portion, demonstrated LD_50_ values below 100 μg/mL, which indicates potential toxicity. The water extract of *P. magnifica* with LD_50_ value of 250 μg/mL also shows potentially harmful effects. Also, an environmental risk resulting from the presence and increasing biomass of potentially toxic benthic cyanobacteria in old colonies should not be underestimated. Toxicity of *Pectinatella* extracts could be partially caused by presence of *Aeromonas* species in material, since we found members of these genera as most abundant bacteria associated with *P. magnifica*. Furthermore, *P. magnifica* seems to be a promising source of certain antimicrobial agents. Its methanolic extract, hexane, and chloroform fractions possessed selective inhibitory effect on some potential pathogens and food spoiling bacteria in the range of MIC 0.5–10 mg/mL. Future effort should be made to isolate and characterize the content compounds derived from *P. magnifica*, which could help to identify the substance(s) responsible for the toxic effects of *P. magnifica* extracts.

## 1. Introduction

*Pectinatella magnifica* (Leidy, 1851) is a colonial fresh-water organism from phylum Bryozoa [1], recently invasive in many areas in Europe and other parts of the world

A colony of *P. magnifica* is formed by a layer of zooids, living on a self-produced jelly blob ranging in weight from a few grams to 10 s of kilograms. Similar to other bryozoans, *P. magnifica* is a filter feeders. They feed mainly on micro plankton and detritus [2]. These organisms reproduce, hibernate, and spread through asexual particles, statoblasts. *P. magnifica* is native to the area east of the Mississippi River, from Ontario to Florida. Its first occurrence recorded outside North America was in Western Europe, in Bille River near Hamburg in 1883 (e.g., [3]). During the 20th century, this species gradually spread across the Elbe river into Germany, Czech Republic, and Poland [4,5]. In France, it was recorded occurring in the area called Franche-Comte in 1994 [5,6]. At present, it occurs also in the Netherlands (its occurrence in the Netherlands was first reported in 2003), in the Rhine basin in the area between Luxembourg and Germany, in Austria, Romania and Turkey [7], Hungary [8], and on the island of Corsica [9]. The newest records of presence are published in Japan and the Korean peninsula [10,11].

The spread in slowly flowing streams is certainly significantly conditioned by the water course [5]. Other possible modes could be spreading thanks to zoochory (statoblasts) on feathers of water birds [11], unharmed statoblasts in the content of stomach in some fish species or water birds [12]. Important for spreading can be human activities [13]. The view of Borg [14] is exceptional in that it does not exclude its cosmopolitan origin.

In related marine bryozoans, the specific bioactive compounds, bryostatins, were identified [15]. They primarily have an anticancer effect [16,17]. Bryostatins belong to the class of alkaloids [15]; furthermore, some isoquinolines, sterols, and some carbohydrates with a heteroatom in structure (nitrophenols or disulfides) were also found in bryozoans. Some of them possess the antibacterial and/or cytotoxic activity [18,19,20]. Except that prevents cell division, some of these metabolites have caused dermatic allergy and have shown antihelmintic activity [21]. Bryostatins are considered to be important promising pharmaceutical substances [17].

Microbial symbionts (e.g., bacteria, cyanobacteria, algae) of bryozoans represent a significant source of potential bioactive compounds [22,23]. For example, bryostatins are produced by the bacterial symbiont *Candidatus Endobugula sertula*, which is present in all life stages of bryozoan *Bugula neritina* [24]. Also, the antimicrobial activity of extracts from marine and freshwater bryozoans including *Pectinatella magnifica* have been demonstrated [18,25,26,27].

It is assumed that the biomass of *P. magnifica* could contain biologically active substances. Therefore, it is important to study this issue, as well as the composition, the quantity, and activity of microbiota of bryozoan colonies. The main aim of our work was to evaluate in vitro toxicity (Section 2.2) and antimicrobial activity of various extracts prepared from *P. magnifica* (Section 2.3). Further, we analyzed the elementary composition of lyophilized *P. magnifica* gel (Section 2.1) and determined toxins of cyanobacteria related to occurrence of *P. magnifica* (Section 2.4).

## 2. Results

### 2.1. Elemental Analysis of P. magnifica Gel

The *P. magnifica* sample for CHN elemental analysis was obtained from a collection of colonies on the pond “Hejtman” in 2014. The gel was mechanically separated from zooids and lyophilized. Elementary analysis showed the composition as 40.0% C, 6.4% H, and 8.7% N.

### 2.2. Cytotoxicity of Extracts

**Figure 1:**
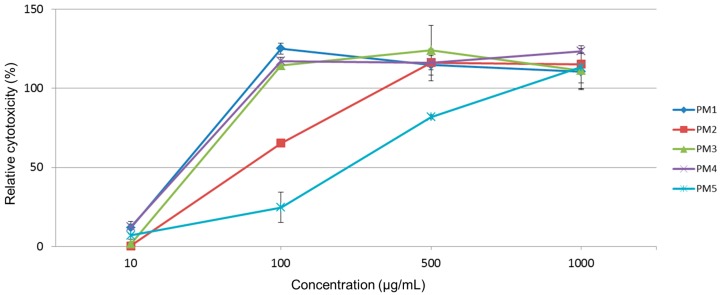
Relative cytotoxicity of *Pectinatella magnifica* extracts in THP-1 cells after 24 h incubation. Legend: PM1—methanolic extract, PM2—hexane portion, PM3—chloroform portion, PM4—ethyl acetate portion, PM5—aqueous portion. The results of LDH release assay are expressed as the means ± SD of three independent experiments, with each condition tested in triplicate.

At all five tested extracts, the cytotoxicity was evaluated as a Relative cytotoxicity (Figure 1), relative to control values (vehicle treated groups). Treatment with *P. magnifica* extracts led to significant toxic effects according to [28] (Table 1) on THP-1 cells, as LD_50_ values were assessed to be <1000 μg/mL. Toxicity expressed as LD_50_ derived from a dose-response curve of the following *P. magnifica* extracts increased as follows: PM5 (aqueous portion, 250 μg/mL) > PM2 (hexane portion, 75 μg/mL) > PM3 (chloroform portion, 40 μg/mL) > PM4 (ethyl acetate portion, 31 μg/mL) > PM1 (methanolic extract, 29 μg/mL).

### 2.3. Antimicrobial Activity of Extracts

Determined MICs of *P. magnifica* extract are showed in Table 2. Only methanolic extract (PM1), hexane (PM2), and chloroform portions (PM3) possessed antibacterial effect against some tested bacteria in the range of MICs from 0.5 to 10 mg/mL. The Gram-positive bacteria were more sensitive to PM1-3 extracts than the Gram-negative. From the Gram-negatives only the growth of *Listeria monocytogenes* ATCC 7644 was inhibited by PM1-3 extracts at MICs 10 mg/mL. The most susceptible bacterium to all three active fractions was potentially pathogenic *Clostridium difficile* CCM 3593. In general, the best results were obtained for chloroform portion (PM3) which inhibits the growth of 10 out of 22 tested strains at the lowest MICs. The hexane portion, inhibiting eight strains, was the second most active substance and methanolic extract affected only four bacterial strains. None of tested bacteria were affected by ethyl acetate and aqueous phase and no growth inhibition caused by DMSO (solvent control) was observed in the control.

Culturable aerobic bacteria were found in the *P. magnifica* colonies in counts of 5.88 ± 0.71 log CFU/g (mean ± S.D., *n* = 8), the numbers varied between 4.96 and 6.71 log CFU/g. More variable were those bacterial counts obtained after anaerobic cultivation. Viability of anaerobes (including facultative anaerobes) was from 2.30 to 6.52 CFU/g, 4.12 ± 1.11 log CFU/g in average. Forty isolates out of 49 selected for detailed identification were satisfactorily classified by MALDI-TOF MS analysis. No reliable results were obtained in nine cases. In 40 strains, a secure genus and probable species identification with score values of 2.000–2.299 were observed. *Aeromonas veronii* was found to be the most abundant species (25 strains) in *Pectinatella* colonies, followed by *Aeromonas hydrophila*, *Aeromonas sorbia* (four strains of both species), *Sphingomonas pituitosa*, and *Lactobacillus plantarum* (one strain each). Five strains were identified only to the genus level, two strains as *Chryseobacterium* spp., and two others as *Herbaspirillum* spp., and one as *Pseudomonas* spp.

Our comparison between bryozoan colonies-associated assemblages and those occurring outside bryozoan colonies showed us that cyanobacteria and algae formed a conspicuous biomass mainly in old colonies. When compared with plankton and periphyton, algae, and cyanobacteria demanding a higher trophic degree prevailed (coccal greens *Desmodesmus* spp., *Chlorococcum* etc.; small diatoms *Stephanodiscus hantzschii*, *Nitszchia* cf. *palea*; and filamentous cyanobacteria *Leptolyngbya*, *Komvophoron*, and *Phormidium* spp.).

### 2.4. Cyanobacterial Toxins Determination

Cyanobacteria were found in *P. magnifica* colony gels (typically of genus *Pseudanabaena*, *Komvophoron*, *Phormidium*, and *Leptolyngbya*) [29]. Their number increases with the colony lifetime as indicated by the inner colony gel color (from red to green). Toxicity of *P. magnifica* occurrence may come from the cyanobacteria which are known as a source of several hepatotoxins, e.g., microcystins (MCs).

Samples were lyophilized biomass (zooids together with the colony gel) and the surrounding water from the location where the colonies were sampled. The results are summarized in Table 3.

## 3. Discussion

Although invasive species are viewed as major threats to ecosystems worldwide, few such species have been studied in detail enough to identify the pathways, magnitudes, and timescales of their impact on native fauna and flora [30]. *P. magnifica* is a new Bryozoa species in Czech Republic with the spread of invasive character [4]. This colonial animal is a filtrator and owing to its massive occurrence it may have an important influence of the ecosystem (species composition, trophic level, hydrochemistry) [31]. This species has not been studied as a key species of any ecosystem yet, and its ecology and chemistry are little known.

Morse [32] has published in 1930 preliminary results about composition of the jelly-like secretion of *P. magnifica* concluding that the gel is not a collagen-like polymer but rather a true protein. Taking into account also an extreme growth rate of the colony gel, he has considered *P. magnifica* as an interesting animal with a rapid proteins production. A course of biuret reaction of the gel has been similar to albumins, the gel has been heat-coagulable, and in the proteins analysis the author has confirmed aminoacids of tyrosine, tryptophan, and cystine. Inorganic compounds of sodium chloride, calcium and, surprisingly, no phosphorus have been confirmed. In statoblasts, the author has presumed the presence of common chitin since, after hydrolysis, glucosamine and galactosamine have been found.

Biomass development in recent seasons 2005–2015 in the Třeboňsko area (South Bohemia, Czech Republic) and monitoring of MC–RR, MC–LR, and nodularins (an HPLC method with the limit of detection of 0.2 mg/L) were already reported [33].

### 3.1. Elemental Analysis of P. magnifica Gel

*P. magnifica* forms huge jelly colonies, composed from material, which has still not been chemically well elucidated. Although the name “Pectinatella” resembles pectin molecules as basic building blocks, it seems that the name more probably comes from the physico-chemical properties of the jelly colony. The CHN elementary composition (40.0% C, 6.4% H, and 8.7% N) shows, that the analyzed material is a mixed composition of protein and polysaccharide, or a strongly glycosylated protein, as pure proteins usually show twice higher content of nitrogen. It can be speculated that the compound(s) that form the colony gel should be relatively simple compound(s) considering the zooid/colony size (2–4 mm/200–400 mm), sources of nutrition (fresh-water), and the enormous growth rate (≈0.5 m in diameter/month).

On the other hand, the analyzed material could be partially contaminated by presence of bacteria, cyanobacteria, and algae, as described further. Chemical composition of the colony gel and the zooid layer is under examination.

### 3.2. Cytotoxicity Tests

Since in related marine bryozoans (phylum Bryozoa) the specific bioactive or toxic compounds were identified [15], we aimed to evaluate cytotoxic effects of the *P. magnifica* lyophilized material. Based on our previously published results [34,35] we used THP-1 cell line as a model system to detect cytotoxic effects of extracts prepared from *P. magnifica*. Results of the toxicity assay showed us that all samples of evaluated *P. magnifica* extracts—except aqueous phase—demonstrated LD_50_ values below 100 μg/mL, which indicate to potential toxicity. The water extract of *P. magnifica* with LD_50_ value of 250 μg/mL also shows potentially harmful effects. Since the behavior of *Pectinatella* exerts signs of invasive character, its potential direct or environmental toxicity should be further studied. However, the tests carried out by our group are using the leukemic THP-1 cells and more test on normal non-cancer cells should be carried out to verify or to refute the toxicity.

### 3.3. Antimicrobial Activity

Extracts of marine [18] and other species of freshwater bryozoans [25] including *P. magnifica* [26,27] have previously been tested to determine their antimicrobial activities. In correspondence with the need of discovering new potentially antimicrobial natural products, bacteria inhibiting activity of *P. magnifica* extracts described in this study, may be considered as promising. The highest antibacterial effect of *P. magnifica* methanolic, hexane, and chloroform extracts was observed against *Clostridium difficile*, bacterium known as the pathogen responsible for various intestinal disorders [36]. Also, other potential pathogens (*Clostridium perfringens*, *Staphylococcus aureus*) and food spoiling bacteria (*Bacillus cereus*, *Listeria monocytogenes*) were inhibited by tested extracts. Recently also Pejin et al. [26] determined antimicrobial activity of crude extracts of *P. magnifica*. In contrast to our experiment, tested bacteria were inhibited not only by methanolic, hexane, and chloroform extract but also by ether and aqueous fractions. *Salmonella*, *Escherichia*, and enterococci tested in our study were resistant to *P. magnifica* extracts whilst Pejin et al. [26] demonstrated effect against these bacteria. *S. aureus*, *B. cereus*, *Micrococcus* spp., *L. monocytogenes*, and *Pseudomonas aeruginosa* were sensitive to *Pectinatella* extract in both studies but MIC determined in our experiment were much higher (2–10 mg/mL) compared to Pejin et al. [26], who demonstrated MIC lower than 1 mg/mL in all cases. The differences in MICs values could be caused by different content of active compounds in *Pectinatella* extract due to dissimilar microbial colonization, environment composition, and methods of extract preparation. Similarly to our experiments, Pejin et al. [25] described inhibition effect of hexane fraction from freshwater *Hyalinella punctata* against *B. cereus*, *L. monocytogenes*, and *S. aureus*. In contradiction with our results, hexane fraction from *Hyalinella punctata* was active also against *Escherichia coli*, *Pseudomonas aeruginosa*, and *Salmonella enterica Typhimurium*. Antimicrobial activity of extracts from different species of Antarctic bryozoans against *B. cereus* was also reported [18], but at tested ether extracts. Although, MICs determined in our experiments were relatively high—much higher than those usual for antibiotic—it must be taken in account that the mixtures of substances were tested. Further, methanolic extract, hexane, and chloroform portions from *P. magnifica* will be separated, identified, and tested for the antimicrobial activity.

Because contamination with foreign bacteria from surrounding water could not be completely prevented during the sampling for microbiological analysis, an important caveat is that not all cultured bacteria may be host specific or even symbiotic. Identified bacteria had previously been described as ubiquitous in water, soil, plants, and other environmental samples. Zooids situated on the surface of *Pectinatella* colonies possess simple U-shaped gut which is presumably inhabited by described bacteria. We can only speculate, if these genera are part of natural gut microbiota or may be pathogenic for *Pectinatella*. *Aeromonas veronii*, the most frequently isolated bacteria, is species ubiquitous in fresh water and have been found in association with a variety of vertebrates and invertebrates with both beneficial and pathogenic outcomes. They have been reported to cause wound infections and diarrhea in humans and have been found as a pathogen and a gut symbiont in some fishes [37]. Sreedharan et al. [38] demonstrated marked cytotoxic and hemolytic activity, which were responsible for the pathogenic potential of *Ae. veronii* strains. This information may indicate that bacteria colonizing *Pectinatella* may be liable for the toxicity of its extracts. Since, simultaneous cytotoxic and antibacterial activity is common for many substances produced by microorganisms, bacteria associated with bryozoan colonies may be responsible also for the antimicrobial activity of their extracts [39].

Toxicity caused by planktonic cyanobacteria such as *Microcystis* and *Woronichinia* does not appear to be an issue, because of a relatively scarce abundance of these planktonic species in matrix. Nevertheless, an enrichment in the rising biomass of benthic filamentous cyanobacteria (*Leptolyngbya*, *Komvophoron*, less also *Phormidium*), compared with surrounding water, can bring a significant dose of cyanotoxins (produced by them) to environment mainly during decomposition of *Pectinatella* colonies. All these genera include a plethora of species, many of which are known to produce toxins including microcystins (MCs) [40,41]. Benthic cyanobacterial assemblages are commonly formed by a mixture of toxic and non-toxic genotypes and toxin concentrations can be highly variable in space and time [42]. Even in a very small sample (such as 1 cm^2^) of a benthic mat formed by *Phormidium* can coexist both toxic (anatoxins producing) and non-toxic strains [31]. However, at present, it is not possible to distinguish between two plausible mechanisms (direct toxic effect of *P. magnifica* vs. toxicity of symbiotic bacteria) on the basis of the data in this study. Thus, in the nearest future, we put our efforts into the isolation and characterization of content compounds derived from *P. magnifica*, which could help us to identify the substance(s) responsible for the toxic effects of *P. magnifica* extracts.

### 3.4. Cyanobacterial Toxins Determination

Microcystins (MCs) belong to a diverse group of oligopeptides produced by cyanobacteria. Over the last few decades, MC and nodularins have become a serious ecological and health issue due to the massive cyanobacterial water blooms that have developed in eutrophied waters worldwide [43].

The toxicity of and risks from some MC variants have been studied in detail [44,45], and the World Health Organization recommends a provisional guideline 1 µg/L of MC–LR for drinking water [46]. Therefore, we adapted a published LC-MS/MS method [47,48] in order to monitor the most common structural (MC–RR, MC–YR, and MC–LR) variants of these highly toxic compounds.

Cyanobacteria and algae were found both in *P. magnifica* matrix and inner surface (a part of a colony attached to substrate). They can play an important role in production of bioactive compounds [29]. Assemblages associated with bryozoan colonies were compared with those occurring outside bryozoan colonies. Several deductions can be made from data in Table 3: (a) three of the four samples (lines 2–4) exhibit much lower concentrations of toxins in the colony than in the surrounding water, which excludes a significant effect of pre-concentration of cyanobacteria inside the colony; (b) the pond “Hejtman” exhibits higher concentration of MCs than the gravel sandpit “Veselí I”, which can be explained by higher trophicity of pond waters; and (c) values in August exceed those in July which follows a natural growth of the animal biomass. 

MC concentrations (sum of the three major structural variants) in the surrounding water of the two sampling sites ranged between 5–309 ng/L, which is below the WHO limit (1 μg/L), and also relatively lower than the typical concentrations found in the Czech Republic or other countries [49,50]. For example, median concentration of MCs in water samples collected in Czech reservoirs during the summer of 2012 was 970 ng/L, and concentration of microcystins exceeded 300 ng/L in 70% samples [50]. Concentrations of MCs detected in the biomass of *Pectinatella* ranged between 2–52 ng/g d.w., were lower than concentrations typically present in cyanobacterial water blooms, where MC concentrations can reach several mg/g d.w. [49]. However, MCs can be often detected in the environment and biota samples also in ng/g d.w. levels, as reported for cyanobacterium *Gloeotrichia* collected from oligotrophic reservoirs [51], cyanobacteria *Leptolyngbya* and *Geitlerinema* isolated from coral reefs [52]. Similarly, trace concentrations of MCs could be found also in lichen [53] or periphyton samples [54], tissues of aquatic organisms exposed to MCs [55,56], or in freshwater sediments [43].

## 4. Materials and Methods

### 4.1. Elemental Analysis of P. magnifica Gel

The lyophilized gel was used for determination of elementary composition. The PE 2400 Series II CHNS/O Analyzer (Perkin Elmer, Waltham, MA, USA) was used for simultaneous determination of C, H, and N in the sample. In the CHN operating mode the instrument employs a classical combustion principle to convert the sample elements to simple gases (CO_2_, H_2_O, and N_2_). The PE 2400 analyzer performs automatically combustion and reduction, homogenization of product gases, separation, and detection. A microbalance MX5 (Mettler Toledo, Greifensee, Switzerland) was used for precise weighing of samples.

### 4.2. Cytotoxicity Tests

The biomass of *P. magnifica* was obtained on July and August 2012 at selected locations in Třeboňsko basin (South Bohemia, Czech Republic), samples were rapidly frozen at −30 °C and then lyophilized. Extracts from *P. magnifica* lyophilisate were prepared in the Department of Natural Drugs, Faculty of Pharmacy, University of Veterinary and Pharmaceutical Sciences Brno, Czech Republic. Lyophilized *P. magnifica* biomass (980 g) was extracted in 70% methanol (3 × 24 h, PM1). The extract was dissolved in 90% methanol and extracted with hexane (3 × 1 L) to get (after removal of hexane by rotavapor) hexane portion (15 g, PM2). The methanol part was diluted with water to 20% methanol solution and extracted with chloroform (3 × 1 L). Chloroform was removed from chloroform portion using rotavapor to get 3 g of PM3. The water residue was consequently extracted with ethylacetate (3 × 1 L) to obtain (after removal of ethylacetate using rotavapor) 10 g of PM4. The water from the water portion was removed via lyophilization to yield 26 g of PM5.

RPMI 1640 culture media, phosphate buffered saline (PBS), and antibiotics (penicillin and streptomycin) were purchased from Lonza (Verviers, Belgium). Fetal bovine serum (FBS) was purchased from (PAA Laboratories, Pasching, Austria). All other reagents were from Sigma-Aldrich (St. Louis, MO, USA).

The human THP-1 cell line was purchased from the European Collection of Cell Cultures (Salisbury, UK; Methods of characterization: DNA Fingerprinting (Multilocus probes) and isoenzyme analysis). Cells were cultured in RPMI 1640 medium supplemented with antibiotics (100 U/mL penicillin, 100 mg/mL streptomycin), 10% FBS, and 2 mM l-glutamine. Cultures were kept in an incubator at 37 °C in a water-saturated 5% CO_2_ atmosphere in air. Cells were passaged at approximately one-week intervals. Cells were free from mycoplasma infection (Hoechst 33258 staining method).

Cytotoxicity of *P. magnifica* extracts was determined using a LDH assay kit (Roche Diagnostics, Mannheim, Germany) as described previously [34]. THP-1 cells were exposed for 24 h at 37 °C to various extract concentrations ranging from 10 to 1000 μg/mL in RPMI 1640 medium (concentrations were selected according to [57]. For LDH assays, cells were seeded into 96-well plates (5 × 10^4^ cells/well in 100 μL culture medium) in triplicate in serum-free RPMI 1640 medium and measurements were taken 24 h after the treatment with the compounds. The median lethal dose values, LD_50_, were deduced through the production of a dose-response curve. The maximum concentration of DMSO in the assays never exceeded 0.1%. All data from three independent experiments were evaluated using GraphPad Prism 5.00 software (GraphPad Software, San Diego, CA, USA, www.graphpad.com).

### 4.3. Antimicrobial Activity

All five *P. magnifica* extracts were tested for their antimicrobial activity against bacteria listed in Table 1. Tested bacteria (potential pathogens, food-spoiling psychrophilic bacteria, and intestinal bacteria) were subcultured in appropriate media and conditions for 24 h prior to the test. A broth microdilution method was used to determine minimal inhibition concentrations (MICs) of the PM1-5 extracts. All the *P. magnifica* extracts were diluted in dimethyl sulfoxide (DMSO), its concentration did not exceed 1% (a concentration inhibiting bacterial growth). Two-fold dilutions were carried out, starting from an initial concentration of 20 mg/mL, employing 96-well microtiter plates. The bacterial inoculum was standardized to achieve density of 1 × 10^6^ CFU/mL. For the anaerobes (clostridia and bifidobacteria), the plates were prepared in an anaerobic chamber (Bugbox, BioTrace, Bridgend, UK) and then incubated for 48 h at 37 °C in an anaerobic jar (Anaerobic Plus System, Oxoid, Basingstoke， UK). Plates were prepared and cultivated under aerobic condition for the rest of tested bacteria. The turbidity in the wells was determined spectrophotometrically using a Microplate reader Infinite M200 (Tecan Trading AG, Männedorf, Switzerland) at 420 nm to evaluate the growth. MICs were defined as the lowest concentration of a *P. magnifica* extract inhibiting growth of the tested bacteria by ≥80% compared to the control. The tests were done in triplicates.

Eight *Pectinatella* samples for microbiota analysis were aseptically collected, transferred to vials containing oxygen-free peptone water, kept in a refrigerator and analyzed within five hours after collection. Samples were serially diluted in the Wilkins-Chalgren broth (Oxoid, Basingstoke, UK) under anaerobic condition. Appropriate dilutions were transferred to Petri dishes and immediately filled with yeast extract-tryptone agar (YT; Oxoid) supplemented with 1 g/L of glucose. Plates were incubated under both anaerobic condition (Anaerobic Plus System; Oxoid) at 25 °C for five days and aerobic condition at the same temperature for three days.

After incubation, six colonies representing a wide range of pigments and colony types were picked up from each sample and cultivation variant. Bacteria were enriched in YT medium and identified. A total of 96 isolates were evaluated for their purity and morphological characteristics using phase contrast microscopy and Gram staining. Forty-nine pure strains with different morphologies were used for detailed identification by Matrix Assisted Laser Desorption/Ionization Time of Flight Mass Spectrometry (MALDI-TOF MS, Bruker Daltonik GmbH, Leipzig, Germany) using the MALDI BioTyper (TM) system (Bruker Daltonik GmbH, Germany) according to [58]. The obtained raw spectra were analyzed using BioTyper software (version 2.0, Bruker Daltonik GmbH, Leipzig, Germany).

### 4.4. Cyanobacterial Toxins Determination

#### 4.4.1. Sample Preparation

A sample of water (500 mL) was lyophilized, the residues dissolved in 1 mL of 50% methanol/water, then vortexed for 1 min, transferred into an Eppendorf vial, and finally centrifuged (10 min at 21,255× *g*). The supernatant was collected, filtrated (0.45 µm nylon filter) into an HPLC vial, and directly analyzed. Lyophilized biomass was weighed (cca 0.1 g) and transferred into a 15 mL centrifuge vial.

A biomass extraction step started with addition of 3 mL of methanol to 0.1 mg of freeze-dried biomass. The mixture was vortexed for 2 min, sonicated by a sonication needle (Bandelin Sonopuls HD2070 with MS72 probe, 3 × 20 s, cycle 0.9 × 10%, power 70%–75%) and then centrifuged (15 min at 3095× *g*). The supernatant was taken and filtrated (0.45 µm nylon filter) into a 12 mL glass vial. Then the whole process of extraction was repeated (starting with addition of 3 mL of methanol) and the second supernatant portion was combined with the first one. Then the mixture was diluted with 110 mL of MilliQ water (the organic phase ratio approximately 5%).

A solid phase extraction column (Oasis HLB, 60 mg) was first conditioned with 2 mL of methanol by the means of the vacuum manifold, and then equilibrated with 2 mL of MilliQ water. The biomass extract prepared according to previous steps was applied onto the column. Prior the elution, the column was washed with 2 mL of 20% methanol, and the analytes were finally eluted with 2 mL of methanol. Then the solvent of the extract was evaporated to dryness under a nitrogen flow at 45 °C. Finally, the sample was diluted with 200 μL of 50% methanol and vortexed for 1 min, transferred into an Eppendorf vial, and centrifuged (10 min at 21,255× *g*). The supernatant was filtrated (0.45 µm nylon filter) and transferred into an HPLC vial.

#### 4.4.2. MC Determination

The analytical method was based on a protocol suitable for MC detection in very complex matrices such as animal tissues [47]. An Agilent 1260 Infinity liquid chromatograph was used with quaternary pump, thermostatted autosampler, and column oven (Agilent Technologies Inc., Waldbronn, Germany). The analytical column was an Agilent POROSHELL 120 EC-C18 (Agilent Technologies Inc., Santa Clara, CA, USA) (4.6 × 50 mm, 2.7 µm) and Guard column (2.1 × 5 mm, 2.7 µm). The injection volume was 5 µL and the flow rate was set to 0.35 mL/min. The mobile phase (A) was 0.1% formic acid in water, and the mobile phase (B) was 0.1% formic acid in acetonitrile. Gradient conditions were 70% of (A), ramped to 45% (A) over 5 min, then decreased to 10% of (A) from 5.01 and held until 7.0 min. Under these conditions, the retention time was 3.5 min for MC–RR, 4.7 and 4.9 min for MC–YR and MC–LR, respectively.

An Agilent 6460 triple quadrupole mass spectrometer (Agilent Technologies Inc.) with an electrospray ionization (ESI) source was used in positive mode: a nitrogen gas temperature 300 °C and gas flow 5 L/min, nebulizer pressure 45 psi, sheath gas temperature 350 °C and flow 11 L/min, capillary voltage 4000 V, and nozzle voltage 500 V. The dwell time of each compound was 200 ms. Transition *m/z* monitored in LC-MS/MS with multiple reaction monitoring (MRM) mode, collision (CE) and fragmentation energy (FE): MC–RR 520 → 135 (CE 60, FE 203), MC–YR 1046 → 135 (CE 61, FE 130), and MC–LR 996 → 135 (CE 68, FE 206). Matrix effect was compensated by external sample calibration.

Instrumental limit of detection was 0.1 ng/mL, 0.3 ng/mL, and 0.1 ng/mL for MC–RR, MC–YR, and MC–LR resp., which corresponds to method limit of detections 0.2 (MC–RR), 0.6 (MC–YR), and 0.2 (MC–LR) ng/g d.w. or ng/L.

## 5. Conclusions

Since a typical elemental composition of proteins is 56% (carbon), 7% (hydrogen), and 14% (nitrogen) resp., the hypothesis of Morse [32] about true protein composition of PM gel can be falsified. Lower content of carbon and nitrogen found (40.0% and 8.7% resp.) rather suggests admixture of carbohydrates that typically exhibit 40% of carbon (and no nitrogen), so in the PM gel we can presume presence of highly glycosylated protein(s). Also, composition of PM biomass was studied earlier [33]. Two types of chemical compounds were isolated identified from *P. magnifica*: (a) derivatives of fatty acids: myristic acid, pentadecanoic acid, palmitic acid, margaric acid, and stearic acid; and (b) derivatives of sterols: campesterol, cholesterol, stigmasterol, crinosterol, and 7-oxo-sterol.

Hepatotoxic peptides of cyanobacterial origin (MCs) were found in *P. magnifica* samples, but only at levels not exceeding the concentrations typically found in aquatic biota. However, only trace concentrations of MCs were present in the surrounding water, and their levels did not correlate with MC content in bryozoan samples. Thus, cyanobacteria present in *Pectinatella* colonies might be the source of MCs detected in the biomass, rather than contamination by residues of the surrounding water during the sampling.

We have also been monitoring MC content in PM biomass for the last years with a HPLC method reported previously [33]. However, the method employs a UV detector—i.e., it exhibits a higher LOD = 1.3 mg/kg d.w. On the other hand, this LOD in dry biomass corresponds to a real concentration of MC in water 0.13 ng/L [33]. Until now we have observed no sample that would overstep the limit. Our initial findings of MCs in the biomass of *P. magnifica* indicated probably for the first-time possible biosynthesis of hazardous cyanotoxins by cyanobacterial species colonizing the bryozoan biomass. Further research should focus on isolation and identification of cyanotoxin-producing species and strains, as well as on detailed characterization of seasonal and spatial dynamics of MCs concentration in the bryozoans and in the surrounding water. This would provide not only information relevant for assessment of ecological and human health risks of bryozoan-associated cyanotoxin contamination, but possibly contribute also to our understanding of the ecophysiological role of cyanotoxin production, its impact on the host species or its eventual role in the host-guest chemical interactions.

Based on the results from Section 2.2 and Section 2.3, it can be concluded that all tested *P. magnifica* extracts, except PM5 (aqueous phase), can be considered as potentially toxic. Methanolic (PM1) and ethyl acetate (PM4) extracts showed the highest cytotoxicity; conversely aqueous phase may be regarded as potentially harmful. From these results, further studies on *P. magnifica* should aim its potential negative effects to the environment. It may be assumed that cytotoxic compounds detected in *Pectinatella* extracts could be produced by *Aeromonas*, since the members of these genera were found as the most abundant bacteria associated with *P. magnifica*.

Although, MICs of *P. magnifica* extracts determined in our experiments were relatively high, bacteria inhibiting activity, may be considered as promising, because the mixtures of substances were tested. Further, methanolic extract, hexane, and chloroform portions from *P. magnifica* could be identified and tested for the antimicrobial activity.

An environmental risk resulting from the presence and increasing biomass of potentially toxic benthic cyanobacteria in old colonies should not be underestimated. On the other hand, since the methanolic extract, hexane, and chloroform fractions possessed selective antibacterial effects, *P. magnifica* may be considered as a promising source of antimicrobial substances.

## Figures and Tables

**Table 1:** Classification of cytotoxicity for natural ingredients [28].

Category	IC_50_
Potentially very toxic	IC_50_ < 10 μg/mL
Potentially toxic	10 μg/mL < IC_50_ < 100 μg/mL
Potentially harmful	100 μg/mL < IC_50_ < 1000 μg/mL
Potentially non-toxic	IC_50_ ˃ 1000 μg/mL

**Table 2:** Antimicrobial activities of *P. magnifica* extracts.

Bacterial Strains	Minimal Inhibition Concentrations (mg/mL)		
	PM 1 *	PM 2	PM 3
*Micrococcus luteus* ATCC 10240	10	4	4
*Acinetobacter parvus* CCM 7030	>20	>20	>20
*Bacillus cereus* CCM 2010	5	4	2
*Bifidobacterium bifidum* DSMZ 20215	10	10	5
*Clostridium difficile* CCM 3593	1	0.5	0.5
*Clostridium perfringens* CCM 4435	>20	5	10
*Clostridium perfringens* DSMZ 11778	>20	5	2
*Enterobacter aerogenes* CCM 7797	>20	>20	>20
*Enterococcus faecalis* DMND	>20	>20	>20
*Escherichia coli* DMND	>20	>20	>20
*Escherichia coli* O45 IS	>20	>20	>20
*Escherichia coli* O55 IS	>20	>20	>20
*Lactobacillus brevis* CCM 3805	>20	>20	>20
*Listeria monocytogenes* ATCC 7644	10	10	10
*Moraxella canis* CCM 4590	>20	>20	>20
*Propionibacterium acnes* DSMZ 1893	>20	>20	>20
*Pseudomonas aeruginosa* CCM 1960	>20	>20	10
*Salmonella enterica Enteritidis* ATCC 13076	>20	>20	>20
*Salmonella enterica Typhimurium* IS	>20	>20	>20
*Salmonella* sp. DMND	>20	>20	>20
*Serratia marcescens* DSMZ 30121	>20	>20	>20
*Staphylococcus aureus* ATCC 25923	>20	10	5

* PM1—methanolic extract, PM2—hexane portion, PM3—chloroform portion. ATTC—American Type Culture Collection, CCM—The Czech Collection of Microorganisms, DMND—The Culture Collection of the Department of Microbiology, Nutrition, and Dietetic of the Life Sciences University Prague, DSMZ—German Resource Centre for Biological Material, IS—The strains provided by Ass. Prof. Ing. Igor Šplíchal, CSc.

**Table 3:** Microcystin (MC) determination, the values are concentrations determined in ng/g d.w. for biomass and ng/L for surrounding water.

Location, Sampling Date *	MC–RR	MC–YR	MC–LR	MC–RR	MC–YR	MC–LR
	*P. magnifica* Colony (ng/g d.w.)			Surrounding Water (ng/L)		
Veselí I, 23.7.2015	31.5	6.6	13.9	1.9	<0.6	3.1
Veselí I, 6.8.2015	6.1	<0.6	10.6	38.3	17.9	70.4
Hejtman, 23.7.2015	4.8	<0.6	7.4	6.8	4.5	31.7
Hejtman, 6.8.2015	4.0	<0.6	2.6	121.6	27.5	159.5
Hejtman, 6.8.2015	11.3	6.1	21.9	n.a.	n.a.	n.a.
Hejtman, 9.10.2012	1.7	<0.6	1.4	n.a.	n.a.	n.a.
Hejtman, 31.10.2012	0.7	<0.6	1.4	n.a.	n.a.	n.a.
Hejtman, 9.10.2012	<0.2	<0.6	<0.2	n.a.	n.a.	n.a.
Hejtman, 9.10.2012	<0.2	<0.6	<0.2	n.a.	n.a.	n.a.

* Location were selected from two monitored places in Třeboňsko area (South Bohemia, CZ): a gravel sandpit (Veselí I) and a pond (Hejtman); MC–RR is microcystin RR, MC–YR is microcystin YR, MC–LR is microcystin LR. Values 0.2, 0.6, and 0.2 ng/g d.w. or ng/L, resp. refers to limit of detections of the respective analytical methods (see 4.4), n.a. stands for “not available”.
